# Associations Between Temporomandibular Disorder‐Related Symptoms, Psychological Distress, and Sleep Quality Among Hospital Healthcare Staff

**DOI:** 10.1002/cre2.70428

**Published:** 2026-10-18

**Authors:** Xiang Bian, Guoqiang Yan

**Affiliations:** ^1^ Department of Stomatology Beijing Xiaotangshan Hospital Beijing China; ^2^ Beijing Open University Beijing China

**Keywords:** health personnel, psychological stress, sleep, temporomandibular disorders

## Abstract

**Objectives:**

To examine associations between temporomandibular disorder (TMD)‐related symptoms, psychological distress, and sleep quality among hospital healthcare staff, and to explore whether cumulative psychological–sleep burden was associated with pain‐related TMD in a graded pattern.

**Materials and Methods:**

This cross‐sectional study included 193 healthcare employees from a tertiary public hospital in Beijing, China. TMD‐related symptoms were assessed using self‐reported symptom items from the Diagnostic Criteria for Temporomandibular Disorders (DC/TMD) Symptom Questionnaire for symptom screening rather than definitive clinical diagnosis. Psychological distress was measured using the Depression Anxiety Stress Scales–21 (DASS‐21), and sleep quality was assessed using the Pittsburgh Sleep Quality Index (PSQI). An exploratory psychological–sleep composite burden index was constructed by summing standardized depression, anxiety, stress, and global PSQI scores. Multivariable logistic regression models evaluated associations between the composite index and pain‐related TMD, adjusting for age group, sex, and occupational role. Quartile‐based trend and sensitivity analyses were performed.

**Results:**

Pain‐related TMD was reported by 67 participants (34.7%). Each 1‐SD increase in the composite index showed a positive but borderline association with pain‐related TMD (adjusted OR = 1.36, 95% CI: 1.00–1.86, *p* = 0.052). In quartile‐based analysis, each higher burden quartile was associated with increased odds of pain‐related TMD (adjusted OR = 1.37, 95% CI: 1.03–1.81, *p* = 0.030). After excluding headache‐only cases, the quartile‐based association remained significant (adjusted OR = 1.37, 95% CI: 1.02–1.85, *p* = 0.037). Symptom‐specific analyses showed heterogeneous patterns.

**Conclusions:**

Higher cumulative psychological–sleep burden was associated with greater odds of pain‐related TMD among hospital healthcare staff, particularly in quartile‐based analyses. These findings support a cautious biopsychosocial interpretation and suggest that psychological distress and sleep quality may be relevant considerations in occupational oral health assessment.

## 1 Introduction

Temporomandibular disorders (TMD) represent a group of conditions characterized by functional abnormalities of the temporomandibular joint (TMJ) and masticatory muscles, commonly presenting as joint noises, preauricular pain, limited mandibular movement, and headache (Schiffman et al. 2014). Beyond structural alterations, TMD—particularly painful manifestations—has increasingly been conceptualized within a biopsychosocial framework, in which biological, psychological, and behavioral factors interact in the development and persistence of symptoms (Fillingim et al. 2013; Wan et al. 2025).

Epidemiological studies have reported a prevalence of approximately 25%–30% in the general population (Fillingim et al. 2013; Valesan et al. 2021). Psychological distress—including anxiety, depression, and stress—has been identified as an important factor associated with both the onset and chronification of TMD symptoms (Kindler et al. 2012). Psychological factors may influence masticatory muscle activity, alter central pain modulation pathways, and reduce pain thresholds, thereby contributing to symptom amplification and maintenance (Bair et al. 2003).

Sleep disturbances, which frequently coexist with psychological distress, have also been implicated in TMD. Poor sleep quality has been associated with increased pain sensitivity, impaired descending inhibitory control, and delayed musculoskeletal recovery (Al‐Jewair et al. 2021). Experimental and clinical evidence suggests that disrupted sleep and negative emotional states may converge on shared neurobiological pathways involved in affective and nociceptive processing, potentially reinforcing pain‐related symptoms (Al‐Jewair et al. 2021; Wan et al. 2025).

Within the broader chronic pain literature, increasing attention has shifted from examining isolated risk factors toward integrated models emphasizing cumulative burden. The cumulative risk perspective proposes that simultaneous exposure to multiple psychological and behavioral stressors may exert amplified effects on stress‐regulatory and neuroendocrine systems, thereby increasing vulnerability to chronic pain conditions (Fillingim et al. 2013; Wan et al. 2025). In the context of TMD, previous studies have largely examined anxiety, depression, or sleep disturbance separately (Kindler et al. 2012; Al‐Jewair et al. 2021), whereas evidence regarding the combined effects of psychological and sleep‐related burden on pain‐related TMD manifestations remains limited.

Healthcare staff constitute a particularly relevant occupational group for such investigation. Sustained occupational stress, irregular work schedules, and high psychosocial demands have been associated with adverse mental health outcomes in this population (Wei et al. 2023). These occupational exposures may contribute to elevated psychological distress and sleep disturbance, which in turn could influence susceptibility to TMD‐related complaints.

Therefore, the present study aimed to examine symptom‐specific associations between TMD‐related complaints, psychological distress, and sleep quality among hospital healthcare staff. In addition, we explored whether cumulative psychological–sleep burden was associated with pain‐related TMD symptoms after accounting for basic demographic and occupational role differences. Rather than presenting the association between psychological distress, poor sleep, and TMD‐related symptoms as entirely novel, this study extends this question to hospital healthcare staff, a clinically relevant occupational group. In addition, by incorporating occupational role into the analysis and using an exploratory standardized psychological–sleep composite burden index, this study examines cumulative psychological–sleep burden within an occupational oral health context.

## 2 Methods

### 2.1 Study Design and Participants

This cross‐sectional study was conducted among healthcare staff employed at a tertiary public hospital in Beijing, China. Eligible participants included physicians, nurses, and non‐clinical administrative staff who had been working at the hospital for at least 1 year.

Inclusion criteria were: (1) age between 20 and 60 years; (2) full‐time employment for ≥ 1 year; (3) ability to complete the questionnaires independently; and (4) provision of written informed consent. The age range of 20–60 years was selected to reflect the main working‐age population of active hospital staff in the study setting. Individuals younger than 20 years are generally not regular hospital employees and would require additional ethical considerations, whereas those older than 60 years are more likely to be retired or to have different occupational exposure patterns. Age was collected in predefined categories rather than exact years.

Exclusion criteria included: (1) history of severe psychiatric disorders; (2) previous maxillofacial trauma or temporomandibular joint surgery; (3) current or recent treatment for TMD; (4) long‐term use of medications affecting psychological status or sleep; and (5) pregnancy or lactation.

The study was approved by the Ethics Committee of Beijing Xiaotangshan Hospital (Approval 2025‐07).

### 2.2 Assessment of TMD‐Related Symptoms

TMD‐related symptoms were assessed using self‐reported symptom items from the Diagnostic Criteria for Temporomandibular Disorders (DC/TMD) Symptom Questionnaire (Schiffman et al. 2014). The questionnaire was administered as a self‐report instrument and was not intended to establish a definitive clinical DC/TMD diagnosis, which requires standardized clinical examination. Rather, the symptom items were used to identify TMD‐related complaints for epidemiological analysis. Participants reported whether they had experienced the following symptoms during the past 12 months: preauricular pain, headache, joint clicking, limited mouth opening, and joint locking. Responses were coded as absent = 0 and present = 1. For joint clicking, limited mouth opening, and joint locking, responses indicating unilateral symptoms were coded as symptom present. Responses of “not sure” were treated as missing for symptom‐specific analyses. The symptom items used in this study are provided in Supporting Information: Table S1.

### 2.3 Definition of the Pain‐Related TMD Symptom Outcome

The pain‐related TMD symptom outcome was defined as the presence of preauricular pain and/or headache. Because headache is not specific to TMD and may introduce outcome misclassification, two sensitivity analyses were performed: first, using preauricular pain alone as a more specific pain‐related outcome; second, excluding participants who reported headache without preauricular pain.

### 2.4 Assessment of Psychological Distress and Sleep Quality

Psychological distress was evaluated using the Chinese version of the Depression Anxiety Stress Scales–21 (DASS‐21) (Lovibond and Lovibond 1995), comprising depression, anxiety, and stress subscales. Sleep quality was assessed using the Chinese version of the Pittsburgh Sleep Quality Index (PSQI) (Buysse et al. 1989). Higher PSQI scores indicated poorer sleep quality.

The DASS‐21 contains three 7‐item subscales assessing depression, anxiety, and stress. In the dataset, item responses were recorded on a 1–4 scale; therefore, 7 points were subtracted from each subscale total to recode scores to the standard 0–3 item scale. The corrected subscale scores were then multiplied by 2 according to standard DASS‐21 scoring procedures. Higher scores indicate greater psychological distress.

Depression, anxiety, and stress positivity were defined as scores above the normal range, using the conventional DASS‐21 severity cutoffs: depression ≥ 10, anxiety ≥ 8, and stress ≥ 15.

The PSQI global score ranges from 0 to 21, with higher scores indicating poorer sleep quality. Poor sleep quality was defined as PSQI global score > 5.

The Chinese version of the DASS‐21 has shown acceptable psychometric properties in Chinese‐speaking populations and Chinese hospital workers (Taouk et al. 2001; Jiang et al. 2020), and the Chinese version of the PSQI has been validated in Chinese populations (Liu et al. 1996; Tsai et al. 2005).

### 2.5 Construction of the Psychological–Sleep Composite Burden Index

The composite psychological–sleep burden index was constructed as an exploratory cumulative burden measure rather than a validated diagnostic scale. Depression, anxiety, stress, and PSQI global scores were first standardized into z‐scores to place the four components on a comparable scale. The standardized scores were then summed with equal weighting. Equal weighting was chosen because no established clinical weighting scheme exists for combining DASS‐21 subscales and PSQI scores in TMD‐related symptom research, and because the aim was to capture overall cumulative psychological–sleep burden rather than to assign causal priority to individual components. To improve interpretability, the composite index was analyzed per 1‐standard‐deviation increase and in quartiles. Correlations among the component variables and alternative models including individual components were examined as supplementary analyses and are presented in Supporting Information: Tables S5 and S7, respectively.

### 2.6 Statistical Analysis

Statistical analyses were performed using Jamovi (version 2.6.45). Categorical variables were summarized as frequencies and percentages, and continuous variables as mean ± standard deviation or median and interquartile range, as appropriate.

For the primary analysis, multivariable logistic regression was conducted to examine the association between the psychological–sleep composite burden index and pain‐related TMD. Adjusted odds ratios (ORs) and 95% confidence intervals (CIs) were calculated. The primary fully adjusted model included age group, sex, and occupational role. Age group was entered as an ordinal covariate because age was collected in predefined categories. Sex and occupational role were entered as categorical covariates.

To improve interpretability, the composite burden index was analyzed per 1‐standard‐deviation increase. Quartile‐based analyses were also performed by entering composite burden quartiles as an ordinal variable to evaluate graded associations with pain‐related TMD. Exploratory polynomial trend terms were additionally examined.

Occupational role differences in pain‐related TMD, psychological distress, poor sleep quality, and composite burden index were examined using chi‐square tests or one‐way ANOVA, as appropriate. Sensitivity analyses were performed using preauricular pain alone as the outcome and after excluding headache‐only cases.

Symptom‐specific logistic regression analyses were considered exploratory. Full results, including non‐significant associations, were reported in supplementary tables to avoid selective reporting. No formal correction for multiple comparisons was applied; therefore, symptom‐specific results should be interpreted cautiously. A two‐sided *p*‐value < 0.05 was considered statistically significant.

## 3 Results

### 3.1 Participant Characteristics

A total of 193 healthcare staff were included. The sample included 45 males (23.3%) and 148 females (76.7%). Occupational roles included physicians (*n* = 79, 40.9%), nurses (*n* = 34, 17.6%), and nonclinical administrative staff (*n* = 80, 41.5%). Age was reported in predefined categories, with the largest groups being 31–35 years (*n* = 43, 22.3%) and 36–40 years (*n* = 40, 20.7%). Pain‐related TMD was reported by 67 participants (34.7%). Depression positivity, anxiety positivity, stress positivity, and poor sleep quality were observed in 44 (22.8%), 40 (20.7%), 28 (14.5%), and 84 (43.5%) participants, respectively. Baseline characteristics are presented in Table 1. The prevalence and missing data for individual TMD‐related symptoms and comparisons across occupational roles are provided in Supporting Information: Tables S2 and S4, respectively.

**Table 1:** Participant characteristics (*n* = 193).

Variable	Total sample, *n* = 193
Sex	
Male	45 (23.3%)
Female	148 (76.7%)
Age group, years	
20–25	10 (5.2%)
26–30	19 (9.8%)
31–35	43 (22.3%)
36–40	40 (20.7%)
41–45	26 (13.5%)
46–50	10 (5.2%)
51–55	33 (17.1%)
56–60	12 (6.2%)
Occupational role	
Physician	79 (40.9%)
Nurse	34 (17.6%)
Nonclinical administrative staff	80 (41.5%)
Depression positive	44 (22.8%)
Anxiety positive	40 (20.7%)
Stress positive	28 (14.5%)
Poor sleep quality, PSQI > 5	84 (43.5%)
Pain‐related TMD	67 (34.7%)

*Note:* Age was collected in predefined categories. Depression, anxiety, and stress positivity were defined using DASS‐21 cutoffs of depression ≥ 10, anxiety ≥ 8, and stress ≥ 15. Poor sleep quality was defined as PSQI global score > 5.

Abbreviations: DASS‐21, Depression Anxiety Stress Scales–21; PSQI, Pittsburgh Sleep Quality Index; TMD, temporomandibular disorder.

### 3.2 Composite Burden Index

The composite psychological–sleep burden index had a mean of approximately 0.00 ± 3.47, a median of −1.09, an interquartile range from −2.53 to 1.66, and a range from −3.98 to 15.85 (Supporting Information: Table S3). DASS‐21 subscales were strongly intercorrelated (*r* = 0.833–0.862, all *p* < 0.001), whereas PSQI global score showed moderate correlations with DASS‐21 subscales (*r* = 0.460–0.551, all *p* < 0.001; Supporting Information: Table S5).

### 3.3 Association Between Psychological–Sleep Composite Burden and the Pain‐Related TMD Symptom Outcome

In the fully adjusted model including age group, sex, and occupational role, each 1‐SD increase in the composite index showed a positive but borderline association with pain‐related TMD (adjusted OR = 1.36, 95% CI: 1.00–1.86, *p* = 0.052; Table 2). This finding suggests a positive association between cumulative psychological–sleep burden and the odds of reporting pain‐related TMD, although the association was borderline statistically significant. Female participants also had higher odds of pain‐related TMD compared with males (adjusted OR = 2.84, 95% CI: 1.21–6.67, *p* = 0.016).

**Table 2:** Fully adjusted logistic regression analysis of the association between psychological–sleep composite burden and pain‐related TMD symptom outcome (*n* = 193).

Variable	Adjusted OR	95% CI	*p*‐Value
Composite burden index, per 1‐SD increase	1.36	1.00–1.86	0.052
Age group, per category increase	0.92	0.79–1.08	0.320
Female vs. male	2.84	1.21–6.67	0.016
Nurse vs. physician	2.15	0.91–5.08	0.083
Non‐clinical administrative staff vs physician	1.83	0.91–3.69	0.092

*Note:* Model adjusted for age group, sex, and occupational role. Age group was entered as an ordinal covariate. ORs are presented for the presence of the pain‐related TMD symptom outcome.

Abbreviations: CI, confidence interval; OR, odds ratio; TMD, temporomandibular disorder.

### 3.4 Graded Association Across Quartiles

The prevalence of pain‐related TMD was 27.1%, 33.3%, 31.3%, and 46.9% across quartiles Q1–Q4, respectively. In the fully adjusted ordinal quartile model, each higher quartile was associated with increased odds of pain‐related TMD (adjusted OR = 1.37, 95% CI: 1.03–1.81, *p* = 0.030; Table 3). When linear, quadratic, and cubic polynomial trend terms were entered simultaneously, none reached statistical significance (Supporting Information: Table S8).

**Table 3:** Quartile‐based analysis of psychological–sleep composite burden and the pain‐related TMD symptom outcome (*n* = 193).

Composite burden quartile	*n*	Pain‐related TMD *n* (%)	
Q1 (lowest burden)	48	13 (27.1%)	
Q2	48	16 (33.3%)	
Q3	48	15 (31.3%)	
Q4 (highest burden)	49	23 (46.9%)	
Fully adjusted logistic regression model			
Predictor	Adjusted OR	95% CI	*p*‐Value
Per one‐quartile increase	1.37	1.03–1.81	0.030

*Note:* The upper section shows the distribution of pain‐related TMD across composite burden quartiles. The lower section shows the fully adjusted binary logistic regression model with composite burden quartile entered as an ordinal predictor. The model was adjusted for age group, sex, and occupational role. ORs are presented for the presence of the pain‐related TMD symptom outcome.

Abbreviations: CI, confidence interval; OR, odds ratio; TMD, temporomandibular disorder.

### 3.5 Sensitivity Analyses

When preauricular pain alone was used as a more specific outcome, the associations remained directionally consistent but did not reach conventional statistical significance. The continuous composite index showed an adjusted OR of 1.08 (95% CI: 0.99–1.18, *p* = 0.088), and the quartile‐based model showed an adjusted OR of 1.33 per higher quartile (95% CI: 1.00–1.78, *p* = 0.054). After excluding participants with headache only, the continuous composite index showed a positive but borderline association (adjusted OR = 1.09, 95% CI: 1.00–1.19, *p* = 0.063), and the quartile‐based association remained significant (adjusted OR = 1.37, 95% CI: 1.02–1.85, *p* = 0.037).

### 3.6 Symptom‐Specific Analyses

Full symptom‐specific regression results, including non‐significant associations, are provided in Supporting Information: Table S6. In exploratory models including DASS‐21 subscale scores and PSQI global score simultaneously, anxiety score was positively associated with headache and joint clicking, whereas PSQI global score was positively associated with joint locking. Some individual DASS‐21 subscale estimates showed unstable directions, likely reflecting the high intercorrelations among DASS‐21 subscales. Because several symptom‐specific outcomes were uncommon, particularly joint locking, and because multiple symptom‐specific comparisons were performed, these findings should be interpreted cautiously.

## 4 Discussion

This study found that higher cumulative psychological–sleep burden was associated with greater odds of pain‐related TMD among hospital healthcare staff, particularly in quartile‐based analyses. Although the fully adjusted continuous model was borderline statistically significant, the direction of association remained consistent across sensitivity analyses.

The quartile‐based findings provide supportive evidence for a graded association, although this should not be interpreted as evidence of causality because of the cross‐sectional design. Previous research has demonstrated independent associations between anxiety, depression, and TMD symptoms (Kindler et al. 2012; Ekici 2023), as well as between sleep disturbances and orofacial pain (Al‐Jewair et al. 2021). However, these factors have often been examined separately. This approach provides an exploratory occupational oral health perspective by considering psychological distress and sleep quality as a cumulative burden rather than as isolated exposures. By integrating depression, anxiety, stress, and sleep quality into an exploratory composite index, the present study provides a complementary perspective on the cumulative psychological–sleep burden associated with pain‐related TMD. This perspective is consistent with the biopsychosocial framework of TMD (Fillingim et al. 2013; Wan et al. 2025) and with contemporary views that position TMD within the broader chronic pain spectrum (Furquim et al. 2015). The composite index should be interpreted as an exploratory cumulative burden measure rather than a validated clinical scale. The equal weighting approach was used for interpretability and because no established weighting scheme exists for combining DASS‐21 subscales and PSQI in TMD‐related symptom research.

The biopsychosocial model, originally proposed in general medicine and subsequently applied to chronic pain research (Engel 1977; Gatchel et al. 2007), emphasizes dynamic interactions between psychological processes and physiological regulation. Psychological distress may modulate central nociceptive processing and masticatory muscle activity, potentially lowering pain thresholds and amplifying symptom perception (Bair et al. 2003). Neuroimaging evidence further suggests that negative emotional states influence cortical and subcortical pain regulatory networks (Wiech and Tracey 2009). Sleep disturbance represents another important contributor. Experimental sleep restriction has been shown to increase pain sensitivity and impair descending inhibitory control (Finan et al. 2013; Haack et al. 2020). In combination, overlapping stress‐related and sleep‐related mechanisms may contribute to cumulative strain on regulatory systems, conceptually aligned with stress‐adaptation frameworks described in neuroendocrine research (McEwen 1998). Although causal inference cannot be drawn from the present cross‐sectional design, the observed graded association is compatible with the possibility that integrated psychosocial influences may be relevant to pain‐related TMD.

Symptom‐specific analyses showed heterogeneous patterns across individual TMD‐related complaints. In exploratory models, anxiety score was positively associated with headache and joint clicking, whereas PSQI global score was positively associated with joint locking. The association between anxiety and headache is consistent with previous evidence suggesting close links among psychological distress, pain perception, and headache‐related complaints. Female sex was also associated with headache in the symptom‐specific model, which is broadly consistent with prior reports of sex‐related differences in TMD prevalence, pain perception, and potential hormonal influences in TMD and comorbid headache conditions (Bueno et al. 2018; Warren and Fried 2001). In contrast, limited mouth opening was not significantly associated with psychological distress or sleep quality in the exploratory models, suggesting that mechanical or structural factors may play a more prominent role in some functional manifestations of TMD (Schiffman et al. 2014; de Almeida et al. 2023). Overall, these findings support the view that TMD‐related symptoms represent a heterogeneous spectrum rather than a uniform clinical entity (Furquim et al. 2015; Yap et al. 2025). However, because these analyses were exploratory, involved multiple comparisons, and included uncommon outcomes such as joint locking, the findings should be interpreted as hypothesis‐generating rather than confirmatory.

From an occupational perspective, healthcare staff are frequently exposed to sustained work‐related stress and irregular schedules (Wei et al. 2023), which may contribute to elevated psychological distress and sleep disturbance. TMD‐related pain has also been associated with reduced quality of life in affected individuals (Bitiniene et al. 2018). The observed association between cumulative psychological–sleep burden and pain‐related TMD suggests that brief psychological and sleep screening may be useful in occupational oral health assessment and in the evaluation of individuals presenting with orofacial pain. Such integration may facilitate early identification of high‐risk individuals and promote multidisciplinary management strategies (Reese 2003). Occupational role comparisons showed that nurses had the highest prevalence of pain‐related TMD, although between‐group differences did not reach statistical significance. No significant occupational role differences were observed for depression, anxiety, stress, poor sleep quality, or composite burden index.

Several limitations should be acknowledged. The cross‐sectional design precludes causal inference. TMD‐related symptoms were assessed via self‐report rather than comprehensive clinical examination based on full DC/TMD diagnostic procedures (Schiffman et al. 2014), potentially introducing misclassification bias. Although sensitivity analyses were performed to address the non‐specific nature of headache, outcome misclassification cannot be completely excluded. Some subgroup analyses involved small sample sizes, resulting in wide confidence intervals. Residual confounding from unmeasured lifestyle or occupational factors cannot be excluded. The study was conducted in a single tertiary public hospital in Beijing, which may limit generalizability to other healthcare settings or regions. Detailed occupational exposure variables, such as shift work, night duty, workload, working hours, and years of employment, were not collected; therefore, the role of specific occupational factors could not be examined. The composite psychological–sleep burden index was exploratory and has not been externally validated. Finally, symptom‐specific analyses were exploratory and were not adjusted for multiple comparisons. Future longitudinal research is warranted to clarify temporal relationships and to further explore the clinical utility of cumulative psychological–sleep burden assessment in TMD risk stratification.

## 5 Conclusion

In this cross‐sectional study of hospital healthcare staff, higher cumulative psychological–sleep burden was associated with greater odds of pain‐related TMD, particularly in quartile‐based analyses. The findings support a cautious biopsychosocial interpretation of TMD‐related symptoms and suggest that psychological distress and sleep quality may be relevant considerations in occupational oral health assessment. Longitudinal studies with clinical DC/TMD assessment and detailed occupational exposure measures are needed.

## Author Contributions

Xiang Bian was responsible for study conception, study design, data collection, statistical analysis, and manuscript drafting. Guoqiang Yan contributed to study design, data interpretation, and critical revision of the manuscript for important intellectual content. All authors read and approved the final manuscript and take responsibility for the integrity of the work as a whole.

## Funding

The authors have nothing to report.

## Ethics Statement

This study was approved by the Ethics Committee of Beijing Xiaotangshan Hospital (Approval 2025‐07). All participants provided written informed consent prior to participation.

## Conflicts of Interest

The authors declare no conflicts of interest.

## Supporting information


Supporting File

## Data Availability

The data that support the findings of this study are available from the corresponding author upon reasonable request. Due to ethical and privacy considerations involving human participants, the data are not publicly available.
