# Supplementary material for: Associations Between Temporomandibular Disorder‐Related Symptoms, Psychological Distress, and Sleep Quality Among Hospital Healthcare Staff
Source: Clin Exp Dent Res. 2026 Oct 18;12(5):e70428. doi: 10.1002/cre2.70428 (PMC13677088; doi:10.1002/cre2.70428)
Supplement: Supplementary file 1 [file CRE2-12-e70428-s001.docx]

**Supplementary Material**

**Associations Between Temporomandibular Disorder–Related Symptoms, Psychological Distress, and Sleep Quality Among Hospital Healthcare Staff**

# **Supplementary Table S1** **|** TMD-related symptom items and coding rules

| **Symptom domain** | **Self-reported item used in this study** | **Coding rule** | **Handling of uncertain response** |
| --- | --- | --- | --- |
| Preauricular pain | Presence of pain in the preauricular/TMJ region during the past 12 months | No = 0; Yes = 1 | Not applicable / missing if uncertain |
| Headache | Presence of headache during the past 12 months | No = 0; Yes = 1 | Not applicable / missing if uncertain |
| Joint clicking | Presence of TMJ clicking/noise during the past 12 months | No = 0; Yes/left/right = 1 | “Not sure” treated as missing |
| Limited mouth opening | Presence of limited mouth opening during the past 12 months | No = 0; Yes/left/right = 1 | “Not sure” treated as missing |
| Joint locking | Presence of joint locking during the past 12 months | No = 0; Yes/left/right = 1 | “Not sure” treated as missing |

*Note: Items were self-reported symptom screening items based on the DC/TMD symptom domains and were not intended to establish a definitive clinical DC/TMD diagnosis. The original questionnaire was administered in Chinese; English translations of the symptom items are shown.*

# **Supplementary Table S2 | Prevalence and missing data of TMD-related symptoms**

| **TMD-related symptom** | **Present n (%)** | **Absent n (%)** | **Missing n** |
| --- | --- | --- | --- |
| Preauricular pain | 58 (30.1%) | 135 (69.9%) | 0 |
| Headache | 33 (17.1%) | 160 (82.9%) | 0 |
| Joint clicking | 35 (18.4%) | 155 (81.6%) | 3 |
| Limited mouth opening | 21 (11.1%) | 168 (88.9%) | 4 |
| Joint locking | 9 (4.7%) | 182 (95.3%) | 2 |

*Note: Percentages were calculated using available non-missing responses.*

# **Supplementary Table S3 | Descriptive statistics of DASS-21, PSQI, and composite burden index**

| **Variable** | **Mean ± SD** | **Median** | **Q1–Q3** | **Min–Max** |
| --- | --- | --- | --- | --- |
| Depression score | 5.36 ± 7.14 | 4 | 0–8 | 0–42 |
| Anxiety score | 4.65 ± 6.52 | 2 | 0–6 | 0–42 |
| Stress score | 6.87 ± 7.92 | 4 | 0–12 | 0–42 |
| PSQI global score | 5.53 ± 3.35 | 5 | 3–7 | 0–18 |
| Composite burden index | 0.00 ± 3.47 | -1.09 | -2.53–1.66 | -3.98–15.85 |

*Note: DASS-21 subscale scores were corrected to the standard 0–42 scale. The composite burden index was calculated by summing z-scores of depression, anxiety, stress, and PSQI global score.*

# **Supplementary Table S4 | Comparisons across occupational roles**

| **Variable** | **Physicians**  **n = 79** | **Nurses**  **n = 34** | **Non-clinical administrative staff**  **n = 80** | **P value** |
| --- | --- | --- | --- | --- |
| Pain-related TMD | 21 (26.6%) | 16 (47.1%) | 30 (37.5%) | 0.088 |
| Depression positive | 17 (21.5%) | 6 (17.6%) | 21 (26.3%) | 0.569 |
| Anxiety positive | 17 (21.5%) | 6 (17.6%) | 17 (21.3%) | 0.887 |
| Stress positive | 10 (12.7%) | 4 (11.8%) | 14 (17.5%) | 0.606 |
| Poor sleep quality | 34 (43.0%) | 17 (50.0%) | 33 (41.3%) | 0.685 |
| Composite burden index, mean ± SD | -0.18 ± 3.11 | -0.00 ± 3.59 | 0.18 ± 3.78 | 0.809 |

*Note: P values were obtained using chi-square tests for categorical variables and one-way ANOVA for the composite burden index.*

# **Supplementary Table S5 | Correlation matrix among DASS-21 subscales and PSQI global score**

| **Variable pair** | **Pearson r** | **P value** |
| --- | --- | --- |
| Depression–Anxiety | 0.833 | <0.001 |
| Depression–Stress | 0.862 | <0.001 |
| Anxiety–Stress | 0.833 | <0.001 |
| Depression–PSQI | 0.460 | <0.001 |
| Anxiety–PSQI | 0.480 | <0.001 |
| Stress–PSQI | 0.551 | <0.001 |

*Note: Pearson correlation coefficients were calculated using continuous DASS-21 subscale scores and PSQI global score.*

# **Supplementary Table S6 | Full symptom-specific logistic regression results**

| **Outcome** | **Predictor** | **Adjusted OR** | **95% CI** | **P value** |
| --- | --- | --- | --- | --- |
| Preauricular pain | Depression score | 0.93 | 0.84–1.02 | 0.128 |
| Preauricular pain | Anxiety score | 1.05 | 0.95–1.16 | 0.345 |
| Preauricular pain | Stress score | 1.04 | 0.95–1.14 | 0.349 |
| Preauricular pain | PSQI global score | 1.08 | 0.96–1.21 | 0.211 |
| Headache | Depression score | 0.88 | 0.77–1.00 | 0.042 |
| Headache | Anxiety score | 1.17 | 1.03–1.33 | 0.016 |
| Headache | Stress score | 1.03 | 0.92–1.15 | 0.602 |
| Headache | PSQI global score | 1.09 | 0.95–1.26 | 0.212 |
| Joint clicking | Depression score | 0.89 | 0.78–1.01 | 0.064 |
| Joint clicking | Anxiety score | 1.22 | 1.07–1.38 | 0.003 |
| Joint clicking | Stress score | 0.97 | 0.87–1.08 | 0.609 |
| Joint clicking | PSQI global score | 1.06 | 0.93–1.21 | 0.365 |
| Limited mouth opening | Depression score | 1.05 | 0.91–1.20 | 0.517 |
| Limited mouth opening | Anxiety score | 1.01 | 0.88–1.15 | 0.935 |
| Limited mouth opening | Stress score | 0.98 | 0.86–1.11 | 0.766 |
| Limited mouth opening | PSQI global score | 1.00 | 0.85–1.17 | 0.972 |
| Joint locking | Depression score | 1.10 | 0.94–1.29 | 0.246 |
| Joint locking | Anxiety score | 1.07 | 0.90–1.26 | 0.452 |
| Joint locking | Stress score | 0.84 | 0.67–1.04 | 0.116 |
| Joint locking | PSQI global score | 1.41 | 1.08–1.85 | 0.012 |

*Note: Each model included depression, anxiety, stress, PSQI global score, age group, sex, and occupational role. ORs are presented for the presence of each symptom. Symptom-specific analyses were exploratory and were not adjusted for multiple comparisons. Because DASS-21 subscales were strongly intercorrelated, estimates for individual subscales should be interpreted cautiously.*

# **Supplementary Table S7 | Alternative model including individual DASS-21 subscales and PSQI global score**

| **Predictor** | **Adjusted OR** | **95% CI** | **P value** |
| --- | --- | --- | --- |
| Depression score | 0.89 | 0.81–0.99 | 0.033 |
| Anxiety score | 1.07 | 0.96–1.18 | 0.212 |
| Stress score | 1.08 | 0.98–1.18 | 0.122 |
| PSQI global score | 1.06 | 0.95–1.19 | 0.315 |
| Age group, per category increase | 0.91 | 0.76–1.07 | 0.252 |
| Female vs male | 3.04 | 1.26–7.30 | 0.013 |
| Nurse vs physician | 2.32 | 0.93–5.78 | 0.072 |
| Non-clinical administrative staff vs physician | 1.81 | 0.88–3.70 | 0.104 |

*Note: Model adjusted for age group, sex, and occupational role. DASS-21 subscale scores and PSQI global score were entered simultaneously. Because DASS-21 subscales were highly intercorrelated, individual component estimates should be interpreted cautiously.*

# **Supplementary Table S8 | Exploratory polynomial trend analysis across composite burden quartiles**

| **Trend term** | **Coefficient** | **SE** | **P value** |
| --- | --- | --- | --- |
| Linear term | 0.0968 | 0.5115 | 0.850 |
| Quadratic term | -0.0732 | 0.1610 | 0.649 |
| Cubic term | -0.1977 | 0.2398 | 0.410 |

*Note: The polynomial trend terms were exploratory. None of the linear, quadratic, or cubic terms reached statistical significance when entered simultaneously.*
